# Immunoproteasome subunit LMP7 Deficiency Improves Obesity and Metabolic Disorders

**DOI:** 10.1038/srep15883

**Published:** 2015-10-30

**Authors:** Hiroaki Kimura, Fumitake Usui, Tadayoshi Karasawa, Akira Kawashima, Koumei Shirasuna, Yoshiyuki Inoue, Takanori Komada, Motoi Kobayashi, Yoshiko Mizushina, Tadashi Kasahara, Koichi Suzuki, Yusaku Iwasaki, Toshihiko Yada, Patrizio Caturegli, Masafumi Takahashi

**Affiliations:** 1 Division of Inflammation Research, Center for Molecular Medicine, Jichi Medical University, Tochigi, Japan; 2 Division of Integrative Physiology, Department of Physiology, Jichi Medical University, Tochigi, Japan; 3 Department of Mycobacteriology, Leprosy Research Center, National Institute of Infectious Diseases, Tokyo, Japan; 4 Department of Pathology, The Johns Hopkins School of Medicine, USA

## Abstract

Inflammation plays an important role in the development of obesity and metabolic disorders; however, it has not been fully understood how inflammation occurs and is regulated in their pathogenesis. Low-molecular mass protein-7 (LMP7) is a proteolytic subunit of the immunoproteasome that shapes the repertoire of antigenic peptides on major histocompatibility complex class I molecule. In this study, we investigated the role of LMP7 in the development of obesity and metabolic disorders using LMP7-deficient mice. LMP7 deficiency conveyed resistant to obesity, and improved glucose intolerance and insulin sensitivity in mice fed with high-fat diet (HFD). LMP7 deficiency decreased pancreatic lipase expression, increased fecal lipid contents, and inhibited the increase of plasma triglyceride levels upon oral oil administration or HFD feeding. Using bone marrow-transferred chimeric mice, we found that LMP7 in both bone marrow- and non-bone marrow-derived cells contributes to the development of HFD-induced obesity. LMP7 deficiency decreased inflammatory responses such as macrophage infiltration and chemokine expression while it increased serum adiponection levels. These findings demonstrate a novel role for LMP7 and provide new insights into the mechanisms underlying inflammation in the pathophysiology of obesity and metabolic disorders.

Obesity is a major risk factor for insulin resistance, hyperglycemia, dyslipidemia, and hypertension, which are known as metabolic syndrome, and has become a global public health problem. These metabolic disorders increase the risk for cardiovascular disease and type 2 diabetes mellitus, and contribute to increased mortality and morbidity. Although the pathogenesis of obesity is complex and involves various factors, recent studies have revealed that inflammation in adipose tissue is a key pathogenic player^1^,^2^. In fact, an infiltration of macrophages into adipose tissue is seen in animal models of obesity as well as in obese human subjects with metabolic syndrome. These cells are the major source for the production of inflammatory cytokines/chemokines including tumor necrosis factor-α (TNF-α), interleukin (IL)-1β, and C-C motif chemokine ligand 2 (CCL2, also known as monocyte chemoattractant protein-1 [MCP-1]), which in turn recruit inflammatory cells and further promote adipose tissue inflammation. However, it has not been fully understood how inflammation occurs and is regulated in the pathophysiology of obesity.

The immunoproteasome is an alternative form of the constitutive proteasome that is a key component of the proteolytic machinery^3^. When the cells are stimulated by inflammatory stimuli, such as interferon-γ or TNF-α, three subunits of the constitutive proteasome β1, β2, and β5 are replaced with the immunoproteasome catalytic subunits β1i (low-molecular mass protein [LMP] 2), β2i (multicatalytic endopeptidase complex-like [MECL] 1), and β5i (LMP7), respectively. Although it is widely recognized that the immunoproteasome has a critical role in antigen processing to be presented to cytotoxic T cells by for major histocompatibility complex (MHC) class I molecules, recent investigations have demonstrated that the immunoproteasome can exert additional functions independently of antigen presentation. We have previously shown that the immunoproteasome is overexpressed in a mouse model of autoimmune inflammation of the thyroid gland, and that its blockade restores thyroid morphology and function^4^. Furthermore, recent studies showed that LMP7 inhibition using its specific blockade ONX-0194 (previously known as PR-957) inhibits proinflammatory cytokine production and attenuates the development of experimental inflammatory and autoimmune diseases^5^,^6^,^7^. Therefore, we hypothesized that LMP7 is involved in the pathophysiology of obesity and metabolic disorders. To test this hypothesis, we used mice deficient in LMP7 (LMP7^−/−^), and found that LMP7 deficiency attenuated adipose tissue inflammation and improved the development of obesity and metabolic disorders. The findings obtained from this study demonstrate a novel role for LMP7 and provide new insights into the mechanisms underlying inflammation in the pathophysiology of obesity and metabolic disorders.

## Results

### LMP7 deficient mice are resistant to HFD-induced obesity

**Figure 1:**
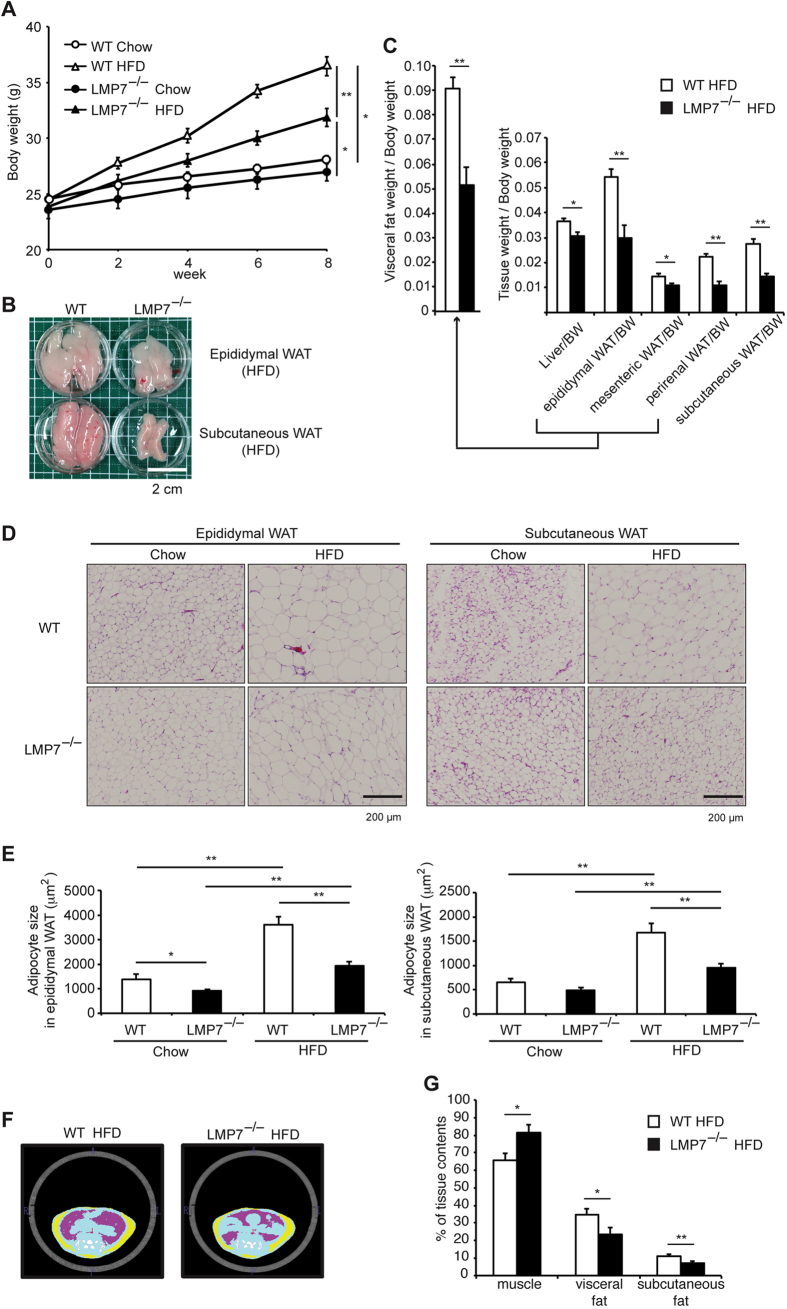
LMP7 deficient mice are resistant to HFD-induced obesity. (**A**) Body weight curves of WT and LMP7^−/−^ mice on normal chow (Chow, n = 8 for each) or HFD (n = 12 for each). (**B**) Representative images of epididymal and subcutaneous WAT in HFD-fed WT or LMP7^−/−^ mice. (**C**) Relative weights (tissue weight/body weight) of HFD-fed WT or LMP7^−/−^ mice (n = 8–12). (**D**) Representative images of hematoxylin and eosin (HE) staining in epididymal and subcutaneous WAT of WT and LMP7^−/−^ mice. (**E**) Quantitative analysis of adipocyte size in epididymal and subcutaneous WAT (n = 8). (**F**) Representative abdominal images of HFD-fed WT or LMP7^−/−^ mice by CT analysis (blue: muscle, pink: visceral fat, yellow: subcutaneous fat). (**G**) Quantitative analysis of muscle, visceral fat, and subcutaneous fat contents (n = 12 for each). Data are expressed as mean ± SEM. *p < 0.05, **p < 0.01.

We first examined whether LMP7 deficiency could influence the development of obesity using wild-type (WT) and LMP7^−/−^ mice fed with normal chow or HFD for 8 weeks. Although there was no significant difference in body weight gain between WT and LMP7^−/−^ mice on normal chow diet, LMP7^−/−^ mice significantly gained less body weight than WT mice on high-fat diet (HFD) feeding (Fig. 1A). The mass of epididymal and subcutaneous white adipose tissue (WAT) was decreased dramatically in LMP7^−/−^ mice, compared to that in WT mice (Fig. 1B). The relative weight (tissue weight/body weight) of each WAT in LMP7^−/−^ mice was less than that in WT mice (Fig. 1C, liver: 15.9% reduction, epididymal: 44.7%, mesenteric: 24.9%, perirenal: 50.7%, and subcutaneous: 47.3%). The reduction was histologically attributable to decreased size of adipocytes in epididymal and subcutaneous WAT (Fig. 1D,E). Computed tomography (CT) analysis showed a smaller mass of visceral and subcutaneous fats in LMP7^−/−^ mice than in WT mice (Fig. 1F,G). These findings indicate that LMP7 deficiency protected against HFD-induced obesity.

### LMP7 deficient mice are resistant to HFD-induced metabolic disorders

**Figure 2:**
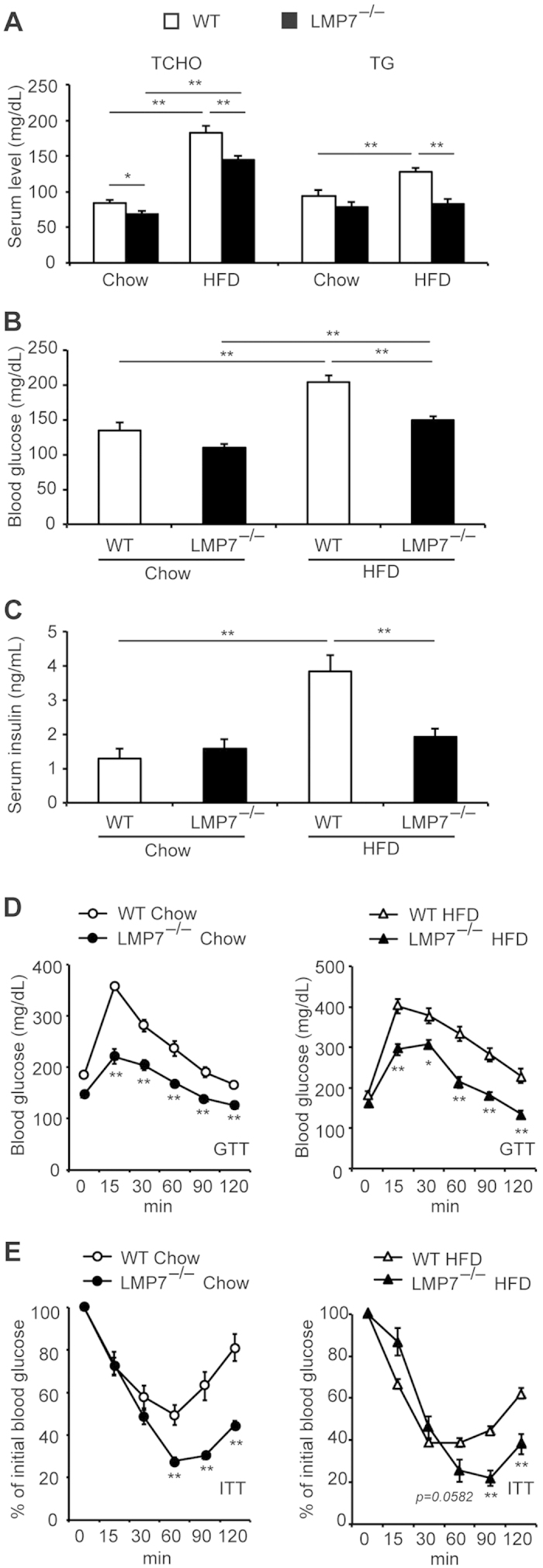
LMP7 deficient mice are resistant to HFD-induced metabolic disorders. (**A–C**) Levels of serum TCHO, TG (**A**), blood glucose (**B**), and serum insulin (**C**) in WT and LMP7^−/−^ mice on normal chow and HFD (n = 8–12). (**D,E**) Results of GTT (**D**) and ITT (**E**) in WT and LMP7^−/−^ mice on normal chow and HFD (n = 8 for each). Data are expressed as mean ± SEM. *p < 0.05, **p < 0.01.

Since obesity leads to metabolic disorders including dyslipidemia and hyperglycemia, we next determined the levels of serum total cholesterol (TCHO) and triglyceride (TG), and blood glucose. The levels of these parameters were considerably increased in WT mice by HFD feeding (Fig. 2A,B), and these levels were significantly decreased in HFD-fed LMP7^−/−^ mice. Consistent with the data on blood glucose levels, serum insulin levels were also elevated by HFD feeding in WT mice and the elevation was significantly suppressed in LMP7^−/−^ mice (Fig. 2C). The glucose tolerance test (GTT) demonstrated better glucose tolerance of LMP7^−/−^ mice compared to WT mice on HFD condition (Fig. 2D). Furthermore, the insulin tolerance test (ITT) showed that LMP7^−/−^ mice improved insulin sensitivity (Fig. 2E). Interestingly, a similar improvement in glucose tolerance and insulin sensitivity was detected in LMP7^−/−^ mice fed with normal chow diet (Fig. 2D,E), indicating that LMP7 is involved in insulin sensitivity independent of obesity. These findings indicate that LMP7 deficiency improved metabolic disorders including lipid and glucose metabolism.

### LMP7 deficiency has no effect on food intake, locomotor activity, and energy expenditure

**Figure 3:**
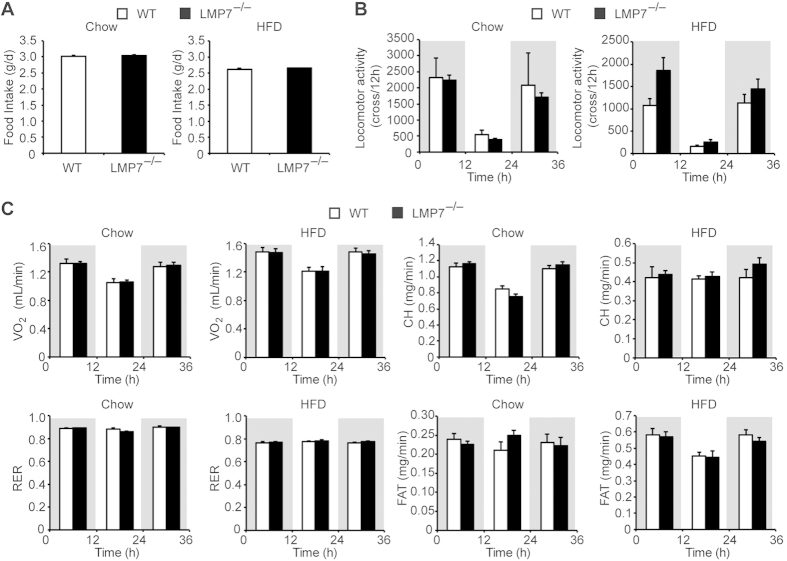
LMP7 deficiency has no effect on food intake, locomotor activity, and energy expenditure. (**A**) Food intake in WT and LMP7^−/−^ mice on normal chow (n = 7–8) and HFD (n = 13–14). (**B**) Locomotor activity in WT and LMP7^−/−^ mice on normal chow and HFD (n = 8 for each). (**C**) Data on energy expenditure. VO_2_, RER, CH, and FAT in WT and LMP7^−/−^ mice on normal chow and HFD (n = 8 for each). Data are expressed as mean ± SEM.

Because food intake was similar between WT and LMP7^−/−^ mice on either normal chow or HFD (Fig. 3A), we measured locomotor activity and energy expenditure in both strain to evaluate differences in basal metabolic rates. There were no significant differences in locomotor activity, oxygen consumption (VO_2_), respiratory exchange ratio (RER), carbohydrate consumption (CH), and fat consumption (FAT) between WT and LMP7^−/−^ mice on either normal chow or HFD feeding (Fig. 3B,C). Supporting this result, although the expression of *Ucp1* (uncoupling protein 1 or thermogenin) was increased in the brown fat tissue by HFD feeding, there was no significant difference of its expression between WT and LMP7^−/−^ mice (data not shown).

### LMP7 deficiency decreases lipid absorption

**Figure 4:**
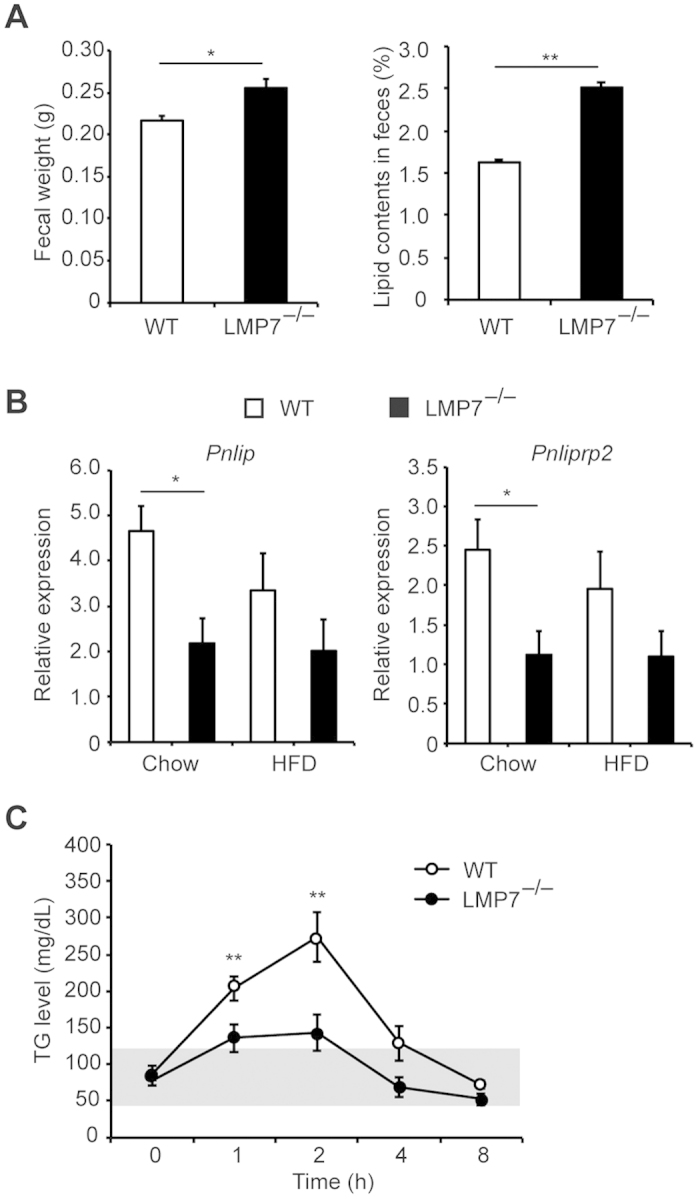
LMP7 deficiency decreases lipid digestion. (**A**) Fecal weights and lipid contents in feces of HFD-fed WT and LMP7^−/−^ mice (n = 8 for each). (**B**) Gene expression of pancreatic lipases (*Pnlip* and *Pnliprp2*) in the pancreas of HFD-fed WT and LMP7^−/−^ mice (n = 4 for each). (**C**) Data on oil load test in WT and LMP7^−/−^ mice. Plasma levels of TG at each time point after oral olive oil administration (n = 6–8). Data are expressed as mean ± SEM. *p < 0.05, **p < 0.01.

Considering no significant difference in food intake, locomotor activity, and energy expenditure between WT and LMP7^−/−^ mice, we assessed the fecal weight and lipid contents. The fecal weight and lipid contents of HFD-fed LMP7^−/−^ mice were significantly higher than those of WT mice (Fig. 4A), suggesting that LMP7 deficiency influences lipid digestion/absorption system. Since fatty acid binding proteins 1 (FABP1) and FABP2 expressed in the intestine have been shown to regulate lipid transport^8^, we assessed expression of *Fabp1* and *Fabp2* in the intestinal mucosa, but observed no significant difference of *Fabp1* and *Fabp2* expression (data not shown). We next determined whether fat-digesting lipases, *Pnlip* (pancreatic lipase) and *Pnliprp2* (pancreatic lipase-related protein 2), are downregulated in the pancreas of LMP7^−/−^ mice and found that the expression of *Pnlip* and *Pnliprp2* was decreased in LMP7^−/−^ mice, compared to WT mice; however, it did not reach statistical significance. To better understand the lipid metabolism in LMP7^−/−^ mice, we performed oral oil load test on fasted mice and found that LMP7^−/−^ mice showed a dramatically decreased TG levels after olive oil administration (Fig. 4C). This is consistent with the result indicating that serum TG level was not elevated in HFD-fed LMP7^−/−^ mice (Fig. 2A). These results indicate that LMP7 deficiency reduced lipid absorption.

### LMP7 in bone marrow and non-bone marrow cells contributes to the development of obesity

**Figure 5:**
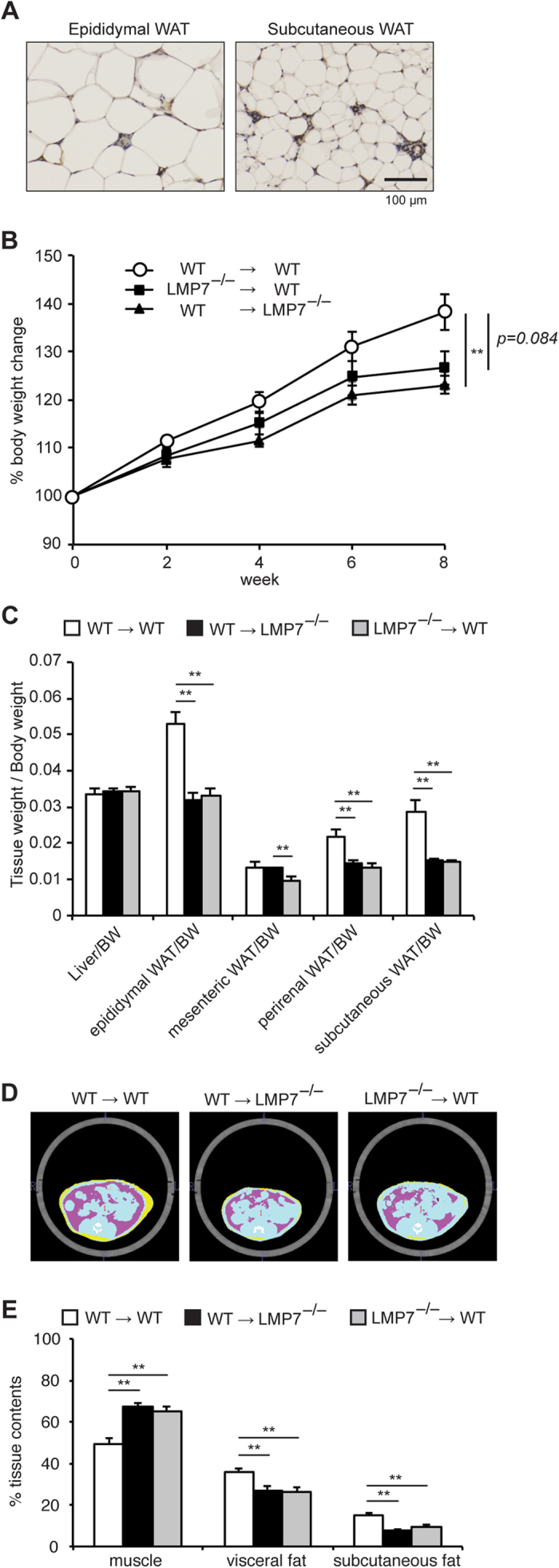
LMP7 in bone marrow and non-bone marrow cells contributes to the development of obesity. (**A**) Representative images of immunostaining for LMP7 in epididymal and subcutaneous WAT of HFD-fed WT mice. (**B**) Body weight change of WT → WT, LMP7^−/−^ → WT, WT → LMP7^−/−^ mice on HFD (n = 10–11). (**C**) Relative weights (tissue weight/body weight) of WT → WT, LMP7^−/−^ → WT, WT → LMP7^−/−^ mice on HFD (n = 10–11). (**D**) Representative abdominal images of WT → WT, LMP7^−/−^ → WT, WT → LMP7^−/−^ mice on HFD by CT analysis (blue: muscle, pink: visceral fat, yellow: subcutaneous fat). (**E**) Quantitative analysis of muscle, visceral fat, and subcutaneous fat contents (n = 10–11). Data are expressed as mean ± SEM. *p < 0.05, **p < 0.01.

The immunoproteasome was expressed predominantly in bone marrow-derived inflammatory cells^5^. Consistent with this, immunohistochemical staining showed that LMP7 was strongly expressed in the stromal vascular area of epididymal and subcutaneous WAT, while it was weakly expressed in adipocytes (Fig. 5A). To determine the contribution of bone marrow-derived LMP7 to the prevention of obesity, we generated 3 types of bone marrow-transferred chimeric (BMT) mice (WT → WT, LMP7^−/−^ → WT, WT → LMP7^−/−^) and evaluated the HFD-induced obesity. The body weight gain and the mass of epididymal and subcutaneous WAT were significantly decreased in WT → LMP7^−/−^ mice, compared with WT → WT mice (Fig. 5B,C). In addition, the body weight gain and the WAT mass also tended to be decreased in LMP7^−/−^ → WT mice although it did not reach statistical significance (*p* = 0.084). These results are further supported by the fat content measurements using CT analysis (Fig. 5D,E). These findings suggest that LMP7 in both bone marrow- and non-bone marrow-derived cells contributed to the development of HFD-induced obesity.

### LMP7 deficiency attenuates inflammatory responses in adipose tissues

**Figure 6:**
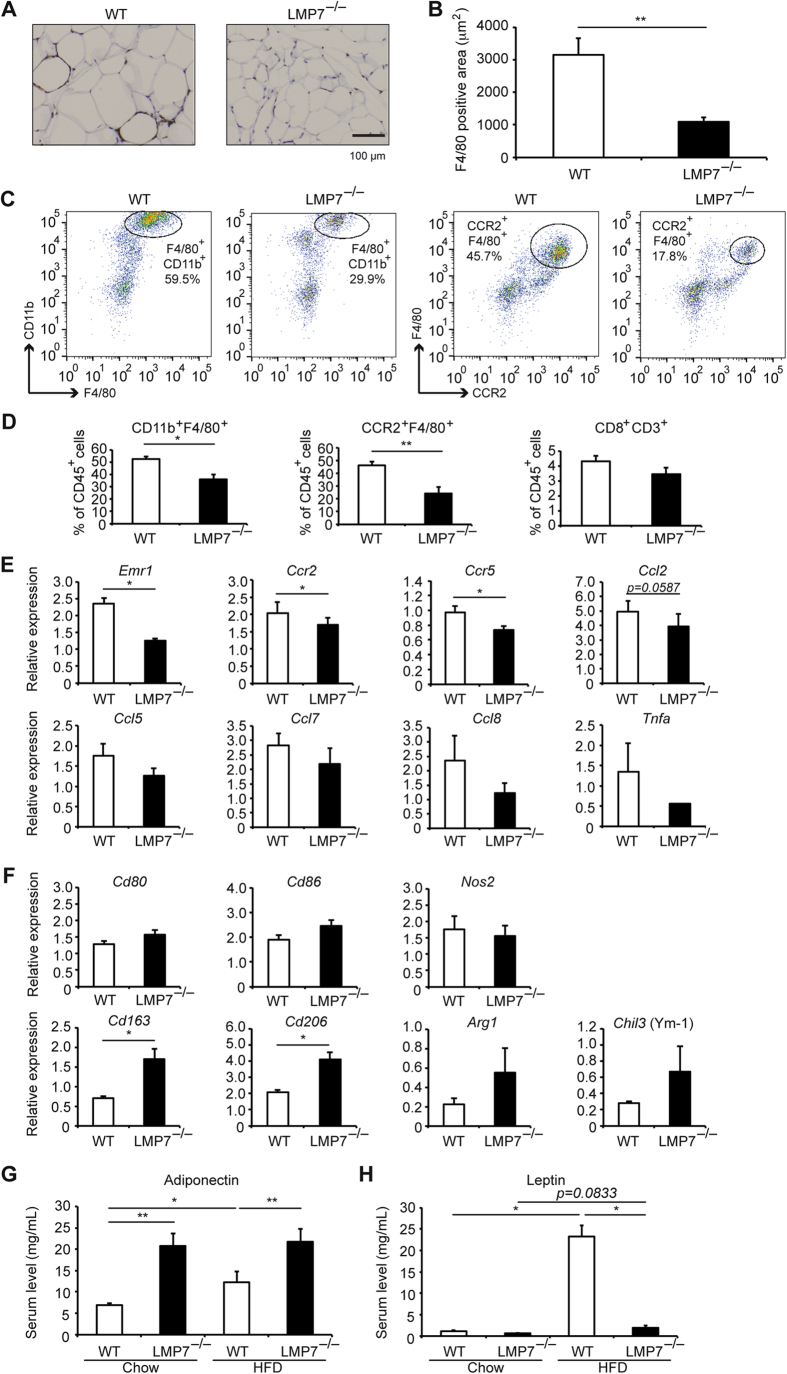
LMP7 deficiency attenuates inflammatory responses in adipose tissues. (**A**) Representative images of immunostaining for F4/80 in epididymal WAT of WT and LMP7^−/−^ mice. (**B**) Quantitative analysis of F4/80-positive areas (n = 9–10). (**C**) Representative images of infiltrated macrophages (CD11^+^F4/80^+^ and F4/80^+^CCR2^+^) in epididymal WAT of HFD-fed WT and LMP7^−/−^ mice by flow cytometry analysis. (**D**) Quantitative analysis of infiltrated macrophages (CD11^+^F4/80^+^ and F4/80^+^CCR2^+^) and CD8^+^ T cells (CD8^+^CD3^+^) in epididymal WAT of HFD-fed WT and LMP7^−/−^ mice (n = 5–7 for each). (**E**) Gene expression in epididymal WAT of HFD-fed WT and LMP7^−/−^ mice (n = 8 for each). (**F**) Expression of macrophage M1 markers (*Cd80, Cd86*, and *Nos2*) and M2 markers (*Cd163, Cd206, Arg1*, and *Chil3*) in epididymal WAT of HFD-fed WT and LMP7^−/−^ mice (n = 4 for each). (**G**,**H**) Serum adiponectin and leptin levels in WT and LMP7^−/−^ mice on normal chow and HFD (n = 8 for each). Data are expressed as mean ± SEM. *p < 0.05, **p < 0.01.

Since infiltration of macrophages in epididymal WAT is a hallmark of obesity^9^,^10^, we assessed infiltration of macrophages in HFD-fed mice. Immunohistochemical analysis using an antibody against F4/80 (macrophage marker) revealed a significant lower number of infiltrated macrophages in epididymal WAT of LMP7^−/−^ mice, compared to WT mice (Fig. 6A,B). Furthermore, flow cytometry analysis showed that the number of inflammatory macrophages (F4/80^+^CD11b^+^ and F4/80^+^CCR2^+^), but not of CD8^+^ T cells (CD8^+^CD3^+^), was significantly decreased in epididymal WAT of LMP7^−/−^ mice, compared to WT mice (Fig. 6C,D). Accordingly, gene expression of *Emr1* (epithelial growth factor-like module containing, mucin-like, hormone-receptor-like 1) encoding F4/80, and *Ccr2* and *Ccr5* (CC chemokine receptors on macrophages) was significantly less in the epididymal WAT of LMP7^−/−^ mice (Fig. 6E). The decreased expression of CC chemokine ligands, including *Ccl2*, *Ccl5*, *Ccl7*, and *Ccl8*, was observed in LMP7^−/−^ mice, but it did not reach statistical significance (Fig. 6E). To analyze the contribution of regulatory T cells, we also examined *Foxp3* expression. Consistent with previous reports^11^, HFD feeding decreased *Foxp3* expression in both WT and LMP7^−/−^ mice (Supplementary Fig. S1). In addition, *Foxp3* expression tended to be higher in LMP7^−/−^ mice than that in WT mice on HFD feeding; however, it did not reach statistical significance. We also assessed the expression of proinflammatory macrophage M1 markers (*Cd80*, *Cd86*, and *Nos2*) and anti-inflammatory macrophage M2 markers (*Cd163*, *Cd206*, *Arg1*, and *Chil3* [also known as Ym1]) in epididymal WAT of WT and LMP7^−/−^ mice. The expression of M2 markers was increased in LMP7^−/−^ mice compared to that of WT mice on HFD feeding, whereas the expression of M1 markers did not differ between WT and LMP7^−/−^ mice (Fig. 6F), suggesting that the switch towards an anti-inflammatory phenotype of macrophages contributes to the phenotypes observed in LMP7^−/−^ mice.

Because inflammatory cytokines are critical factors in the development of metabolic disorders^2^, we assessed serum levels of several inflammatory cytokines. There were no significant differences of serum IL-1β, IL-6, and CCL2 levels between WT and LMP7^−/−^ mice (data not shown). In addition, IL-17 was not detected in serum of WT and LMP7^−/−^ mice. We next examined the effect of palmitate on inflammatory cytokine production in primary macrophages isolated from LMP7^−/−^ mice *in vitro*. Palmitate treatment significantly increased the production of IL-1β and IL-6, but not TNF-α, in WT macrophages (Supplementary Fig. S2). However, the production of IL-1β and IL-6 was markedly inhibited in LMP7^−/−^ macrophages. In addition, palmitate treatment failed to induce TNF-α production.

To investigate the anti-inflammatory effect of LMP7 deficiency in WAT, we finally assessed whether adiponectin and leptin are involved in this process. Serum adiponectin levels were significantly higher in HFD-fed LMP7^−/−^ mice, compared to HFD-fed WT mice (Fig. 6G). Consistently, expression of *Adipoq*, encoding adiponectin, was also upregulated in the epididymal WAT of LMP7^−/−^ mice (Supplementary Fig. S3). The transcription factors encoded by *Pparg* (peroxisome proliferator-activated receptor γ), *Cebpa* (CCAAT/enhancer-binding protein [C/EBP]-α), and *Cebpb* (C/EBP-β) are known to be inducible of *Adipoq* expression^12^,^13^. The expression of all these transcription factors was significantly upregulated in LMP7^−/−^ mice, compared with WT mice (Supplementary Fig S3). Serum leptin levels were significantly increased in WT mice by HFD feeding, and these levels were markedly decreased in HFD-fed LMP7^−/−^ mice (Fig. 6H). These results suggest that adiponectin and leptin were involved in the improvement of adipose tissue inflammation and metabolic disorders in in LMP7^−/−^ mice.

## Discussion

The major findings of this study are as follows: (1) LMP7 deficiency decreased HFD-induced fat accumulation and protected against obesity; (2) LMP7 deficiency improved glucose intolerance and insulin sensitivity, and improved lipid and glucose metabolism (3) there was no difference of food intake, locomotor activity, and energy expenditure between WT and LMP7^−/−^ mice; (4) LMP7 deficiency reduced lipid absorption; (5) BMT experiments showed that LMP7 in both bone marrow- and non-bone marrow-derived cells contributed to the development of HFD-induced obesity; (6) LMP deficiency decreased inflammatory responses such as macrophage infiltration and chemokine expression while it increased adiponection production. The results of the present study suggest that LMP7 contributes to macrophage-driven inflammatory responses in adipose tissue and the development of obesity and metabolic disorders. To our knowledge, this study provides the first evidence that LMP7 plays a major role in the development of obesity and metabolic disorders.

Increasing evidence indicates that inflammation plays an important role in the development of obesity and metabolic disorders^1^,^2^. Although there are a number of reports describing the role of inflammation in their process, it has not been fully understood how inflammation occurs and is regulated. Although the immunoproteasome is known to play an essential role in MHC class I antigen presentation, recent investigations demonstrated LMP7 is required to produce proinflammatory cytokines, such as TNF-α and IL-6, and to progress experimental arthritis and colitis^5^,^6^. In the present study, we demonstrated that LMP7 deficiency inhibits adipose tissue inflammation, obesity, and metabolic disorders: this suggests that LMP7 inhibition has a potential for the prevention and treatment of obesity and metabolic disorders. Indeed, several experimental studies reported that the LMP7 selective inhibitor ONX-0914 is considerably effective in treating experimental arthritis and colitis^5^,^6^. Thus, the effect of this LMP7 inhibitor on obesity and metabolic disorders remains to be examined in future studies.

LMP7 is predominantly expressed in immune and inflammatory cells^5^. Indeed, we observed that LMP7 was mainly expressed in the stromal vascular area of WAT, it is expected that LMP7 has a predominant role in bone marrow-derived inflammatory cells. BMT experiments showed the substantial role of bone marrow LMP7 in the development of obesity. *In vitro* experiments showed that the production of proinflammatory cytokines such as IL-1β and IL-6 is inhibited in LMP7^−/−^ macrophages. Furthermore, LMP7 deficiency promoted M2 polarization of macrophages. All these findings suggest that macrophage LMP7 contributes to the induction of inflammatory responses in adipose tissue. Consistent with this, LMP7^−/−^ mice showed improvement of glucose intolerance and insulin sensitivity. On the other hand, LMP7 deficiency in non-bone marrow cells unexpectedly resulted in a reduction of body weight gain and fat mass. This observation suggests that the resident cells in adipose tissue contribute to the beneficial effects of LMP7 deficiency. In this regard, we found that serum levels of adiponectin and expression of *Adipoq* were significantly increased in LMP7^−/−^ mice. Consistently, the expression of *Pparg*, *Cebpa*, and *Cebpb*, inducible of *Adipoq* expression, was significantly upregulated in LMP7^−/−^ mice. In addition, serum leptin levels were markedly decreased in HFD-fed LMP7^−/−^ mice. Because there is no difference of food intake between WT and LMP7^−/−^ mice, it is likely that leptin resistance can occur in HFD-fed WT mice. Since it is known that both adiponectin and leptin regulate inflammatory and glucose homeostasis^14^, it is likely that that adiponectin and leptin may contribute to the improvement of glucose metabolism and adipose tissue inflammation in LMP7^−/−^ mice.

We showed that LMP7 deficiency decreased the expression of pancreatic lipases, increased fecal lipid contents, and inhibited the increase of plasma TG levels upon oral oil administration or HFD feeding. On the other hand, serum levels of glucose and insulin were not affected in LMP7^−/−^ mice on normal chow diet. Glucose intolerance and insulin sensitivity were improved in LMP7^−/−^ mice. Thus, LMP7 influences external and internal secretory function of the pancreas, and is involved in the digestive and hormonal homeostasis, which would be important in the metabolic status of mice.

Several limitations of this study should be noted. First, although we focused on the role of macrophages, the roles of other inflammatory cells still need to be determined. In this regard, Nishimura *et al.* previously reported that CD8^+^ T cell infiltration precedes accumulation of macrophages in adipose tissue of HFD-fed mice and that adipose CD8^+^ T cells have an essential role in the initiation and propagation of adipose tissue inflammation^11^. Furthermore, they recently reported that regulatory B cell dysfunction contributes to the progression of adipose tissue inflammation in the process of obesity^15^. Second, we found the increased adiponectin production in adipose tissue of LMP7^−/−^ mice; however, the molecular mechanism how LMP7 influences its production remains unclear. Third, it is reported that human *PSMB8* (alias LMP7) mutation causes autoinflammatory diseases, such as Nakajo-Nishimura syndrome, CANDLE syndrome and JMP syndrome^16^,^17^,^18^,^19^. The phenotypes of these autoinflammatory diseases are inconsistent with our findings obtained from LMP7^−/−^ mice. Similar to our findings, several investigators demonstrated that L LMP7^−/−^ mice show decreased expression of inflammatory cytokines and attenuated disease activity in experimental colitis^6^. Furthermore, as mentioned above, treatment with ONX-0914 decreases inflammatory cytokine production and attenuates the development of experimental arthritis or colitis^5^,^6^. In contrast, however, Seifert *et al.* recently revealed that immunoproteasomes are more active than constitutive proteasomes and this enhanced activity of the immunoproteasomes prevents harmful protein aggregation during inflammation^20^. The authors further showed that elevated levels of protein aggregates in the brain of LMP7^−/−^ mice and that the inflammation induced by experimental autoimmune encephalomyelitis is more severe in the absence of LMP7. However, this finding was disputed by Nathan *et al.* who reported that immunoproteasomes did not degrade ubquitinated proteins more efficiently than constitutive proteasomes^21^. At present, although the reason for this discrepancy remains unclear, it is likely that whether LMP7 deficiency exerts a protective or deleterious effect on disease activity may depend on the underlying pathogenesis and/or tissue affected by the disease^22^. Thus, further investigations are necessary to understand the precise role of LMP7 in the development of inflammation-associated diseases.

In conclusion, we clearly showed that LMP7 deficiency inhibits macrophage-driven inflammation in adipose tissue and improve the development of obesity and metabolic disorders in HFD-fed mice. Based on our findings, we assume that LMP7 deficiency inhibits adipose tissue inflammation by inhibiting proinflammatory cytokine induction in macrophages and adiponection produced by adipocytes. Furthermore, LMP7 influences external and internal secretory function of the pancreas. The present study not only demonstrates that LMP7 may be a potential therapeutic target for prevention and treatment of obesity and metabolic disorders but also provides new insights into the mechanism underlying the pathophysiology of these disorders.

## Methods

### Animals

All animal experiments were approved by the Use and Care of Experimental Animals Committee of the Jichi Medical University Guide for Laboratory Animals, and were carried out in accordance with the Jichi Medical University guidelines. C57BL/6J WT mice were purchased from Japan SLC, Inc. (Hamamatsu, Japan). LMP7^−/−^ mice have been previously described^4^. Male mice (9–10 week old) were fed with either 60 kcal% HFD (Research Diets: D12492, Japan LSG, Tokyo) or standard chow (CE-2; CLEA Japan Inc., Osaka). Each mouse was weighed every 2 weeks. At terminal points, mice were fasted for 6 h, weighed again and blood was collected to obtain serum. After perfusion, the tissues were carefully excised and weighed.

### Micro-CT analysis

Micro-CT analysis was performed using LaTheta LCT-200 (Hitachi Aloka Medical, Tokyo, Japan). The mass of muscle, visceral, and subcutaneous fat was analyzed using the LaTheta software (Hitachi Aloka Medical).

### Biochemical test

Blood was collected from the tail vein of 6 h-fasted mice. Blood glucose levels were measured using a Terumo MEDISAFE^TM^ Blood Glucose Meter (Terumo Co., Tokyo, Japan). Serum or plasma levels of TCHO and TG were measured using a FUJI DRI-CHEM system (Fujifilm, Tokyo, Japan). Serum levels of insulin, adiponectin, and leptin were measured using ELISA kits (Sibayagi, Gunma, Japan; R&D Systems Inc., Minneapolis, MN; and BioVendor R&D, Brno, Czech Republic).

### GTT and ITT

GTT was performed in mice on a standard chow or HFD for 8 weeks. Mice were fasted for 6 h beforehand with free access to water. Blood glucose was then measured just before the intraperitoneal glucose injection (1 g/kg body weight in saline) and subsequently at 15, 30, 60, 90, and 120 min post-administration. ITT was performed in a similar fashion by administering an insulin injection (0.75 U/kg body weight) instead of glucose.

### Histology and immunohistochemistry

HE staining and immunohistochemistry for F4/80 and LMP7 were performed as described previously^4^,^23^.

### Real-time RT-PCR analysis

Total RNA was prepared from the kidney using ISOGEN (Nippon Gene Co., Ltd., Toyama, Japan) according to the manufacturer’s instructions. Real-time RT-PCR analysis was performed using the Takara TP960 PCR Thermal Cycler Dice Detection System (Takara Bio Inc, Shiga, Japan) to detect mRNA expression as described previously^23^. Gene expression was normalized using *Actb* (β-actin) expression using the software provided with the system. Primers used for RT-PCR analysis are listed in Supplementary Table S1.

### BMT experiments

BMT mice were generated as described previously^24^. To verify the reconstitution of bone marrow after transplantation by this protocol, we used green fluorescent protein (GFP) mice as donors. Flow cytometry analysis showed that 8 weeks after BMT, peripheral blood cells consisted of more than 90% GFP-positive cells^24^. Using this protocol, we generated 3 types of BMT mice: WT → WT, LMP7^−/−^ → WT, WT → LMP7^−/−^ mice).

### Respiratory gas and locomotor analyses

Mice were housed individually in a metabolic chamber for 48 h to allow them to adapt to the environment and to attain a constant respiratory exchange ratio (RER). Respiratory gas (O_2_ and CO_2_) analysis was performed using an open-circuit metabolic gas analysis system connected directly to a mass spectrometer (Arco2000; ArcoSystem, Chiba, Japan). Oxygen consumption (VO_2_), and carbon dioxide production (VCO_2_) were measured every 5 min for each cage (one mouse). RER was calculated as the ratio VCO_2_/VO_2_. Total carbohydrate consumption (CH) and fat consumption (FAT) were calculated using the stoichiometric equations of Frayn as follows: CH = 4.51 × VCO_2_ – 3.18 × VO_2_ [mg/min], and FAT = 1.67 × (VO_2_ – VCO_2_) [mg/min]. Locomotor activity was determined every 5 min for each chamber (one mouse) by the number of infrared beams broken in both X and Y directions using an activity monitoring system (ACTIMO-100; Shinfactory, Fukuoka, Japan) combined with individual metabolic chambers.

### Collection of feces and extraction of feces lipid

Individually housed HFD-fed mouse feces over 24 h were collected and dried using vacuumed centrifugation. Dried feces were weighed and ground in a mortar. Ground feces were transferred into a glass tube and weighed again. Lipids were extracted using chloroform: methanol (2:1) twice following Folch’s method^25^, and weighed. Lipid contents were calculated as % of ground feces weight.

### Flow cytometric analysis

Infiltrating leukocytes in epidydimal WAT were analyzed by flow cytometry as described previously^11^. The data on flow cytometry was obtained using FACSVerse (BD Biosciences, San Jose, CA) and analyzed using FlowJo software (Treestar, Inc., San Carlos, CA). Reagents used for flow cytometric analysis are listed in Supplementary Table S2.

### *In vitro* experiments

Murine peritoneal macrophages were cultured in RPMI1640 (Sigma-Aldrich, St Louis, MO) supplemented with 10% foetal bovine serum (FBS) and 1% antibiotic–antimycotic (Invitrogen, Carlsbad, CA) using 24-well plates. After 48 h, cells were stimulated with 400 μM palmitate (10% BSA)^26^ for 24 h. IL-1β, IL-6, and TNF-α levels in the supernatants were determined using ELISA kits (R&D Systems Inc.).

### Statistical analysis

Outcomes with normal distribution were analyzed by parametric unpaired *t*-test. Outcomes without normal distribution were analyzed by the Mann–Whitney test, the Kruskal–Wallis test, or longitudinal analysis. All analyses were performed using Stata software, release 13 (Stata Corp., College Station, TX). A *p*-value of *<*0.05 was considered statistically significant.

## Additional Information

**How to cite this article**: Kimura, H. *et al.* Immunoproteasome subunit LMP7 Deficiency Improves Obesity and Metabolic Disorders. *Sci. Rep.* **5**, 15883; doi: 10.1038/srep15883 (2015).

## Supplementary Material

Supplementary Information
