# Supplementary material for: Immunoproteasome subunit LMP7 Deficiency Improves Obesity and Metabolic Disorders
Source: Sci Rep. 2015 Oct 30;5:15883. doi: 10.1038/srep15883 (PMC4626825; doi:10.1038/srep15883)
Supplement: Supplementary Information [file srep15883-s1.pdf]

Immunoproteasome subunit LMP7 Deficiency Improves Obesity and Metabolic Disorders

Hiroaki Kimura[1], Fumitake Usui[1], Tadayoshi Karasawa[1], Akira Kawashima[1], Koumei Shirasuna[1], Yoshiyuki Inoue[1], Takanori Komada[1], Motoi Kobayashi[1], Yoshiko Mizushina[1], Tadashi Kasahara[1], Koichi Suzuki[2], Yusaku Iwasaki[3], Toshihiko Yada[3], Patrizio Caturegli[4], Masafumi Takahashi[1]

[1]Division of Inflammation Research, Center for Molecular Medicine, [3]Division of Integrative Physiology, Department of Physiology, Jichi Medical University, Tochigi; [2]Department of Mycobacteriology, Leprosy Research Center, National Institute of Infectious Diseases, Tokyo, Japan; [4]Department of Pathology, The Johns Hopkins School of Medicine, USA.

Correspondence:

Hiroaki Kimura, PhD or Masafumi Takahashi, MD, PhD

Division of Inflammation Research, Center for Molecular Medicine

Jichi Medical University

3311-1 Yakushiji, Shimotsuke, Tochigi 329-0498, Japan

Tel/Fax: +81-285-58-7446/+81-285-44-5365

E-mail: balhiro33@jichi.ac.jp (HK) or masafumi2@jichi.ac.jp (MT)

Supplementary information

Supplementary Fig. S1. Effect of LMP7 deficiency on Foxp3 expression

Expression of *Foxp3* in epididymal WAT of WT and LMP7$^{-/-}$ mice on normal chow and HFD (n = 4 for each).

Supplementary Fig. S2. Effect of LMP7 deficiency on the production of IL-1β, IL-6, and TNF-α

Peritoneal macrophages were isolated from WT and LMP7$^{-/-}$ mice (n = 4 for each), and treated with or without palmitate for 24 h. The levels of IL-1β, IL-6, and TNF-α in the supernatants were assessed. Data are expressed as mean ± SEM. *$p < 0.05$, **$p < 0.01$.

Supplementary figure S3. Effect of LMP7 deficiency on the expression of Adipoq, Pparg, Cebpa, and Cebpb

Epididymal WAT was extracted from WT and LMP7$^{-/-}$ mice fed with HFD for 8 weeks. *Adipoq*, *Pparg*, *Cebpa,* and *Cebpb* mRNA expression was assessed by real-time RT-PCR analysis (normal chow: n = 4, HFD: n = 8 for each). Data are expressed as mean ± SEM. *$p < 0.05$, **$p < 0.01$.

Supplementary Table S1: Primer List for real-time PCR

Gene Symbol	Forward (5' - 3')	Reverse (5' - 3')
Actb	CCTGAGCGCAAGTACTCTGTGT	GCTGATCCACATCTGCTGGAA
Tnfa	AAGCCTGTAGCCCACGTCGTA	GGCACCACTAGTTGGTTGTCTTTG
Emr1 (F4/80)	CCTGGACGAATCCTGTGAAG	GGTGGGACCACAGAGAGTTG
Ccr2	CCTGCAAAGACCAGAAGAGGG	GAGATGTTGATAGTATGCCGTGGA
Ccr5	CTGGCAAAAAGCTGAAGAGCGT	GCAGCATAGTGAGCCCAGAAT
Ccl2	GGCTCAGCCAGATGCAGTTAAC	GCCTACTCATTGGGATCATCTTG
Ccl5	ACCAGCAGCAAGTGCTCCAA	TGGCTAGGACTAGAGCAAGCAATG
Ccl7	GCTGCTTTCAGCATCCAAGTG	CCAGGGACACCGACTACTG
Ccl8	TGCCTGCTGCTCATAGCTGTC	GACATACCCTGCTTGGTCTGGAA
Foxp3	CTCATGATAGTGCCTGTGTCCTCAA	AGGGCCAGCATAGGTGCAAG
Cd80	GGCAAGGCAGCAATACCTTA	CTCTTTGTGCTGCTGATT
Cd86	TCTCCACGGAAACAGCATCT	CTTACGGAAGCACCCATGAT
Nos2	GGCAGCCTGTGAGACCTTTG	GAAGCGTTTCGGGATCTGAA
Arg1	ACAAGACAGGGCTCCTTTCA	AGCAAGCCAAGGTTAAAGCC
Cd163	TCCACACGTCCAGAACAGTC	CCTTGGAAACAGAGACAGGC
Cd206	CAGGTGTGGGCTCAGGTAGT	TGTGGTGAGCTGAAAGGTGA
Chil3 (Ym-1)	GGGCATACCTTTATCCTGAG	CCACTGAAGTCATCCATGTC
Fabp1	CCATGAACTTCTCCGGCAAGTACC	CTTTGGGTCCATAGGTGATGGTGAG
Fabp2	GTGGAAAGTAGACCGGAACGA	CCATCCTGTGTGATTGTCAGTT
Pnlip	CTGGGAGCAGTAGCTGGAAG	AGCGGGTGTTGATCTGTGC
Pnliprp2	ATGCCTATGGATGTCCGTGGA	TGCCCAGGGCTTGTCATTG
Adipoq	GTCAGTGGATCTGACGACACCAA	ATGCCTGCCATCCAACCTG
Pparg	TGTCGGTTTCAGAAGTGCCTTG	TTCAGCTGGTCGATATCACTGGAG
Cebpa	TTGAAGCACAATCGATCCATCC	GCACACTGCCATTGCACAAG
Cebpb	ACCGGGTTTCGGGACTTGA	CCCGCAGGAACATCTTTAAGTGA
Ucp1	TACCAAGCTGTGCGATGTCCA	GCACACAAACATGATGACGTTCC

Supplementary Table S2: Reagents used for flow cytometric analyses

Reagents	Vender	Clone	Conjugates
CD3	BD Biosciences	145-2C11 (hamster)	FITC
CD8a	BD Biosciences	53-6.7 (rat)	PE
CD11b	eBiosciences	M1/70 (rat)	FITC, PE
CD45	eBiosciences	30-F11 (rat)	FITC, PE, APC
F4/80	eBiosciences	BM8	FITC, PE
CCR2	R&D Systems	475301 (rat)	FITC
CD16/CD32	BD Biosciences	2.4G2 (rat)	N/A (Fc block)
7-AAD	BD Biosciences	N/A	N/A

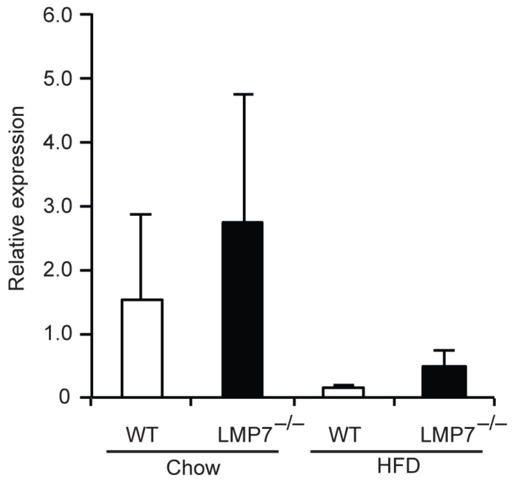
6.0
5.0
4.0
3.0
2.0
1.0
0
Relative expression
WT
LMP7−/−
WT
LMP7−/−
Chow
HFD

Supplementary Figure S2 Kimura H

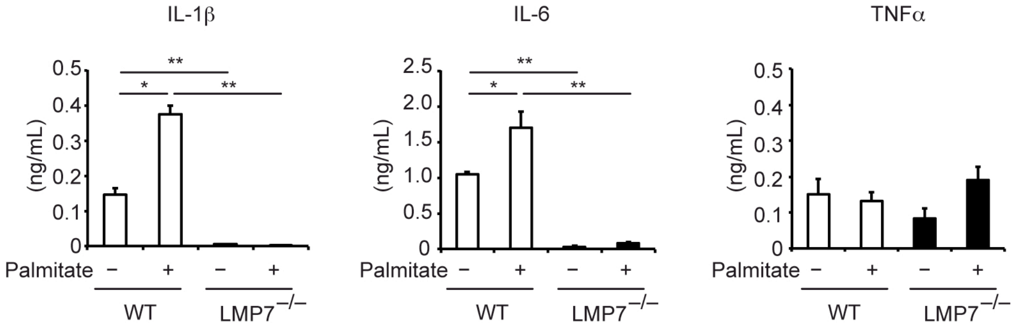

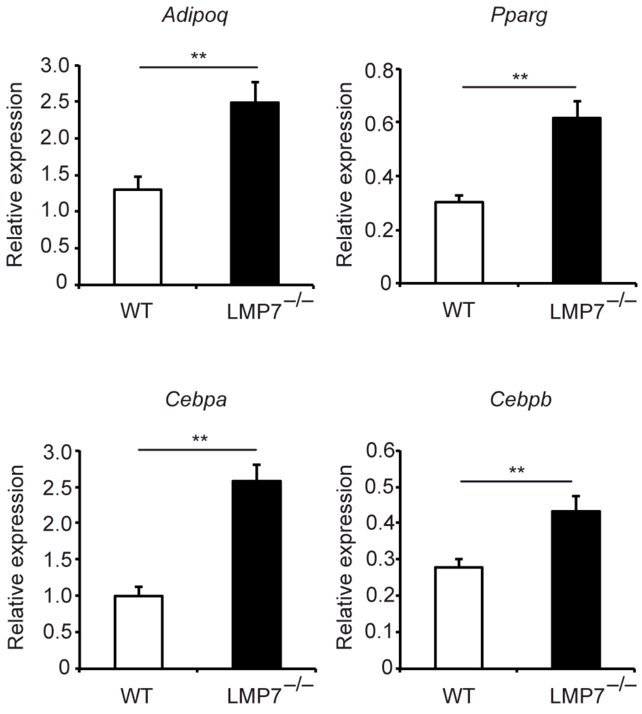

Adipoq
Pparg
Cebpa
Cebpb
Relative expression
**
3.0
2.5
2.0
1.5
1.0
0.5
0
0.8
0.6
0.4
0.2
0.6
0.5
0.3
0.1
WT
LMP7−/−
